# Comparative Evaluation of Two Image-Analysis Software Platforms for Microfluidic Assessment of Red Blood Cell Deformability in Chronic Lymphocytic Leukemia

**DOI:** 10.3390/mi17030389

**Published:** 2026-03-23

**Authors:** Anika Alexandrova-Watanabe, Tihomir Tiankov, Aleksandar Iliev, Ariana Langari, Miroslava Ivanova, Lidia Gartcheva, Margarita Guenova, Emilia Abadjieva, Sashka Krumova, Svetla Todinova

**Affiliations:** 1 Institute of Mechanics, Bulgarian Academy of Sciences, “Acad. G. Bontchev” Str. 4, 1113 Sofia, Bulgaria; anikaalexandrova@abv.bg (A.A.-W.); tiho_bg@abv.bg (T.T.); alxndr.iliev@gmail.com (A.I.); abadjieva@gmail.com (E.A.); 2 Center of Competence for Mechatronics and Clean Technologies “Mechatronics, Innovation, Robotics, Automation and Clean Technologies”—MIRACle, “Acad. G. Bontchev” Str. 4, 1113 Sofia, Bulgaria; arianalangari@abv.bg (A.L.); miroslava.ilieva.ivanova@gmail.com (M.I.); 3 Institute of Biophysics and Biomedical Engineering, Bulgarian Academy of Sciences, “Acad. G. Bontchev” Str. 21, 1113 Sofia, Bulgaria; sashka.b.krumova@gmail.com; 4 National Specialized Hospital for Active Treatment of Hematological Diseases, Zdrave Str. 2, 1756 Sofia, Bulgaria; l.garcheva@hematology.bg (L.G.); m.genova@hematology.bg (M.G.)

**Keywords:** chronic lymphocytic leukemia, Red blood cells, image flow analysis, LabVIEW software, Python software, deformability index

## Abstract

Red blood cell (RBC) deformability is a key determinant of microcirculatory flow and can be altered in hematological disorders such as chronic lymphocytic leukemia (CLL). This study aimed to evaluate RBC deformability under controlled microfluidic flow conditions and to assess the influence of software platform choice on deformability quantification. RBC suspensions from healthy individuals and untreated CLL patients were analyzed using a microfluidic imaging system across a range of shear rates. A dedicated image-processing algorithm was developed and implemented in two software environments (LabVIEW and Python) to automatically detect deformed cells, measure major and minor cell axes, and calculate the deformability index (DI). Both analytical approaches demonstrated a shear-dependent increase in DI in healthy controls, whereas RBCs from CLL patients exhibited reduced deformability and a blunted response to increasing shear rates, particularly at intermediate shear rates. Although LabVIEW produced consistently higher absolute DI values than Python, both platforms showed strong correlation and preserved the same relative trends and group discrimination. These findings demonstrate that microfluidic image flow analysis provides a robust approach for assessing RBC biomechanics and highlight the importance of standardized image-processing workflows for reliable deformability quantification across software platforms.

## 1. Introduction

Red blood cells (RBCs) constitute approximately 45% of total blood volume and are essential for oxygen delivery and carbon dioxide removal. To pass through capillaries narrower than their resting diameter, RBCs undergo significant and reversible deformation. This remarkable deformability of RBCs is determined by the viscoelastic properties of the membrane–cytoskeleton complex, cytoplasmic viscosity, and cells’ unique biconcave geometry [1,2]. The biconcave shape of RBCs is particularly important for deformability, as it minimizes bending energy and facilitates shape adaptation under flow conditions [3]. Alterations in any of these determinants, arising from oxidative and inflammatory stress or other pathological conditions, reduce RBC flexibility, thereby compromising microvascular blood flow, increasing flow resistance, and diminishing tissue oxygen delivery. Consequently, quantitative evaluation of RBC deformability represents a sensitive biomechanical indicator of blood rheological properties and microcirculatory performance [4].

In health, RBC deformability increases under high shear stress and dynamically adapts to flow conditions, facilitating their passage through capillaries. This adaptability allows RBCs to respond to mechanical forces and maintain functional integrity within the microcirculation. However, reduced deformability is associated with a broad range of diseases. Impaired RBC deformation has been reported in metabolic disorders such as diabetes mellitus and sepsis [5], hereditary hemoglobinopathies, malaria infection, and during blood storage, all of which may compromise microvascular perfusion and manifest as clinically relevant rheological dysfunction [4]. Moreover, RBC deformability influences blood viscosity and flow characteristics at both high and low shear rates, thereby affecting perfusion pressures under physiological and pathological conditions [6]. Such alterations often precede overt clinical manifestations, highlighting the potential diagnostic and prognostic value of deformability measurements [6,7]. Therefore, RBC deformability can be significantly altered in systemic inflammatory diseases, and quantifying these changes may provide valuable prognostic information.

Chronic lymphocytic leukemia (CLL) is a hematological malignancy characterized primarily by the accumulation of monoclonal B lymphocytes. However, there is increasing evidence that CLL also affects the rheology and biophysical behavior of RBCs. Systemic inflammation and oxidative stress, which are already detectable in early-stage CLL patients [8], may induce lipid peroxidation and oxidative damage to membrane proteins, leading to spectrin/ankyrin modifications, cytoskeletal remodeling, and increased membrane stiffness of RBCs [9]. These biophysical changes can modify the rheological properties of RBCs, contributing to impaired microcirculation, increased blood viscosity, and an elevated risk of vascular complications. Our recent studies employing microfluidic flow image analysis have shown that RBC aggregation patterns differ between healthy subjects and patients with CLL [10]. These aggregation differences may be related to underlying changes in RBC biophysical properties, particularly deformability, which governs RBC dynamics, cell–cell interactions, and aggregate stability under microvascular shear conditions. Despite their clinical relevance, alterations in RBC deformability in CLL remain insufficiently characterized, and direct measurements in this context are scarce. However, research on leukemic cells has demonstrated increased stiffness of CLL lymphocytes relative to healthy lymphocytes, suggesting that disease-associated systemic biophysical remodeling may extend beyond leukocytes to other circulating blood cells, including RBCs [11].

Microfluidic technologies emerged as powerful tools for assessing RBC deformability under controlled flow conditions that mimic aspects of the in vivo microenvironment. By subjecting cells to defined shear stresses and capturing their deformation responses, these systems provide physiologically relevant measurements of cell mechanics. By mimicking microvascular geometries and applying controlled shear stress, microfluidic platforms enable high-throughput, label-free analysis of individual cells in a dynamic flow environment.

In addition to conventional microfluidic deformability assays, high-throughput microfluidic techniques for mechanical phenotyping have also been developed. A notable example is real-time deformability cytometry (RT-DC), in which cells flowing through narrow microfluidic channels are deformed by hydrodynamic stresses and analyzed using high-speed imaging in real time. This approach enables label-free characterization of cell size, shape, and deformability at very high throughput (hundreds to thousands of cells per second) and has been widely applied for blood and other cell types [12,13,14]. While RT-DC provides rapid, population-level measurements, microfluidic assays like ours offer complementary insights by enabling detailed analysis of individual RBC deformation under precisely controlled shear conditions.

Microfluidic devices allow precise control of flow rates that closely mimic physiological conditions in the microvasculature, generating laminar flow with straight and parallel streamlines near the channel surface. This flow profile facilitates the adhesion of flowing erythrocytes (RBCs) to the channel walls [12]. The application of shear flow induces elongation of RBCs anchored at a single point, accompanied by changes in cell curvature. This deformation results from a balance between hydrodynamic forces and the elastic properties of the RBC membrane, including shear and bending elasticity [13,15].

One of the most widely used indirect measures of RBC elasticity is the change in cell shape, expressed as the Deformability Index (DI = (L_x_ − L_y_)/(L_x_ + L_y_), where L_x_ and L_y_ represent the major and minor axes of an ellipse fitted to the cell contour, respectively [16,17,18]. This parameter reflects the extent of cell elongation under applied mechanical stress and is closely related to other commonly used descriptors, such as the Aspect Ratio or Elongation Index (EI) [4,19,20]. In practice, DI is determined from high-resolution images of RBCs subjected to shear flow, followed by automated contour detection and ellipse fitting to extract the principal geometric axes of the deformed cell [3].

Combined with high-resolution imaging, technology provides a powerful platform for the quantitative assessment of RBC deformability based on dynamic cell behavior [21]. Microfluidic systems offer several advantages over traditional rheological techniques, such as ektacytometry and viscometry, by enabling single-cell resolution, direct visualization, and multiparametric quantification of deformability and morphology [4]. Recent studies have integrated microfluidics with specialized image analysis software [16,19,22] or employed machine learning approaches to automate deformability assessment from cell images, thereby increasing throughput and reproducibility [23]. However, reliable interpretation of such data critically depends on robust image processing algorithms capable of suppressing flow-induced background artifacts and accurately identifying deformed cells.

An additional challenge in image-based deformability analysis is the potential variability introduced by different software environments and implementation strategies. Differences in filtering, segmentation, thresholding, and numerical precision may lead to systematic discrepancies in absolute deformability values, complicating cross-study comparisons and reproducibility. However, the potential impact of different software environments and implementation strategies on the quantification of RBC deformability has received limited attention, particularly in clinically relevant settings such as CLL.

In this study, we present a microfluidic-based approach to quantify RBC deformability using image-processing pipelines implemented in two independent software platforms: LABVIEW (2023 Q3) and Python (3.10). A sine window–based filtering strategy was developed to suppress flow-related background variations while preserving RBC contours. Cell deformation was quantified using a geometrically defined deformability index (DI) derived from ellipsoidal parameters of the cell shape. By applying identical analytical criteria across platforms, we systematically evaluated potential inter-platform variability while maintaining sensitivity to biologically relevant differences in RBC deformability.

Using this framework, we compared RBC deformability in healthy donors and CLL patients under controlled microfluidic flow conditions. Our results demonstrate distinct deformability patterns between the two groups and reveal consistent relative trends across software platforms, despite differences in absolute DI values. These findings highlight both the mechanical alterations of RBCs associated with CLL but also underscore the importance of robust and reproducible image-processing methodologies in obtaining reliable deformability measurements in microfluidic systems.

## 2. Materials and Methods

### 2.1. Study Groups and Ethics Statement

A total of nine untreated patients (mean age 63.9 ± 12.0 years) were included in the study. All patients were diagnosed with CLL at the National Specialized Hospital for Active Treatment of Hematological Diseases, Sofia, Bulgaria. Diagnosis was established according to internationally validated expert recommendations and clinical guidelines of the International Workshop on Chronic Lymphocytic Leukemia (iwCLL) for diagnosis, treatment indication, response assessment, and supportive management of CLL [24,25]. CLL patients with diabetes, renal diseases, autoimmune diseases, or hyperlipidemia were excluded from the study.

The control group comprised 13 healthy individuals (mean age 58.0 ± 7.4 years), with no family history of CLL or other oncohematological diseases.

All participants provided written informed consent before inclusion in the study. The study protocol was approved by the Ethics Committee of the Institute of Biophysics and Biomedical Engineering, Bulgarian Academy of Sciences (permit No. 378ND 26/03/2024) and was conducted in accordance with the Helsinki Principles for Ethical Standards in Research with Human Participation.

### 2.2. Blood Collection and Sample Preparations

Blood samples were collected by venipuncture into two 6 mL tubes (Vacutainer; Becton Dickinson and Company, Franklin Lakes, NJ, USA) containing K_2_EDTA. Blood from CLL patients was collected during their routine hospital visits.

RBC isolation was performed according to the protocol previously described by Alexandrova-Watanabe et al. [26]. Briefly, collected blood samples were centrifuged (Universal 320 R centrifuge, Hettich, Germany), after which the supernatant (plasma and white blood cells) was removed. The RBC fraction was resuspended and washed twice in phosphate-buffered saline (PBS; 140 mM NaCl, 2.7 mM KCl, 8 mM Na_2_HPO_4_, 1 mM KH_2_PO_4_). The hematocrit of the final RBC suspension was adjusted to 40% using a Haematokrit 200 centrifuge (centrifuge Haematokrit 200, Hettich, Germany).

From each blood sample obtained from healthy controls and untreated CLL patients, 10 μL was added to 200 μL of Dextran 70 (Carl ROTH, Karlsruhe, Germany) solution (4 g/dL). This dilution resulted in a final hematocrit of 2% in the RBC suspensions used for all experiments.

### 2.3. Viscosity Measurements

The viscosity of RBC suspensions diluted in Dextran 70 (prepared as described in Section 2.2) was measured under steady-state flow conditions using a Brookfield DV-II+ Pro programmable rotational viscometer (Brookfield Engineering Laboratories, Inc., Middleboro, MA, USA). The instrument was calibrated with water at 37 °C before measurements. The viscosity of the diluted RBC suspensions in Dextran 70, measured at 37 °C, was 1.12 ± 0.04 mPa·s.

### 2.4. Description of Microfluidic System

RBC deformation was investigated using an air-pressure-driven BioFlux microfluidic system (Fluxion Biosciences, Oakland, CA, USA). This system provides a high-quality imaging platform for rheological analyses under flow conditions.

The microfluidic setup consisted of a BioFlux 200 electro-pneumatic flow control pump, a LumaScope 620 inverted fluorescence microscope (Etaluma, San Diego, CA, USA) equipped with a C-Apochromat 10/0.25 NA dry objective (OFN22), BioFlux microfluidic well plates, and a computer workstation running the dedicated specialized control software (LumaView version: 19.07.12) as previously described in Alexandrova-Watanabe et al. (2025) [10]. A schematic illustration of the microfluidic system is provided in the Supplementary Materials (Figure S1).

Cells were visualized using transmitted-light brightfield illumination with phase-contrast imaging. Images were recorded with the system’s sCMOS camera at a resolution of 1600 × 1600 pixels. The spatial calibration of the imaging system corresponded to 0.6 µm per pixel, allowing conversion between pixel units and physical dimensions. To minimize motion blur during flow, an exposure time of 10–20 ms was applied.

RBC deformability assays were performed using BioFlux 24-well plates (0–20 dyn/cm^2^), each containing eight microfluidic channels with cross-sectional dimensions of 350 μm in width and 75 μm in height (rectangular shape).

### 2.5. Design of the Experiments

The microfluidic channels were filled with 200 μL of each prepared RBC suspension diluted in Dextran 70. The suspensions were perfused through the channels at a shear stress of 5 dyn/cm^2^ (corresponding to a shear rate of 446 s^−1^) for 5 min to disperse preexisting RBC aggregates. Thereafter, the flow was abruptly reduced to a low shear stress of 0.1 dyn/cm^2^ (corresponding to a shear rate of 8.9 s^−1^) and maintained for 15 min to promote adhesion of RBCs to the walls of the microchannels.

Increasing shear stresses of 1, 2, 3, 4, 5, 6, and 7 dyn/cm^2^ (corresponding to shear rates of 89, 178, 268, 357, 446, 535, and 625 s^−1^, respectively) were sequentially applied to the microchannels. At each shear rate, RBC elongation increased correspondingly. Once maximum cell elongation was reached (approximately 10 s after applying the respective shear stress), at least five images of the deformed RBCs were captured along the visible length of the channel at randomly selected locations.

### 2.6. Algorithm for Software Image Flow Analysis of RBC Deformability

In this study, an algorithm was developed to evaluate RBC deformability and implemented in two software platforms: LabVIEW (commercial software) and Python (open-source software). Each image of deformed RBCs in the microchannels was processed in parallel using the two developed software programs. The software automatically identified individual cells while excluding RBCs that overlapped or collided with other cells.

LabVIEW workflow: For each selected elongated (deformed) cell, an equivalent ellipse was fitted to the cell contour using a least-squares optimization method. From this fit, the lengths of the major (L_X_) and minor (L_Y_) diameters of an equivalent ellipse, along with the centroid position and orientation angle, were extracted. The cell deformability index (DI) for each cell was calculated as (1):

DI = (L_X_ − L_Y_)/(L_X_ + L_Y_) (1)

Ellipse fitting provides a robust measure of directional deformation, capturing anisotropic elongation while reducing sensitivity to local contour irregularities or noise. The LabVIEW fitting and cell detection were performed using the standard “Fit Ellipse” VI.

Python workflow: The two most distant points belonging to the segmented cell are identified, and the line connecting them defines the primary axis L_X_. This axis determines both the orientation and the maximum size of the cell. All pixels of the cell are then projected onto this axis and onto the perpendicular direction. The extrema of these projections define the dimensions of the smallest enclosing rectangle aligned with L_X_. The length along L_X_ is denoted as L_X_, and the length along the perpendicular direction is denoted as L_Y_.

For each blood sample and at each applied shear rate, all DI values were aggregated, and the mean DI was determined separately by each software program. This standardized pipeline enables systematic comparison of RBC deformability across software platforms while ensuring reproducible quantification of individual-cell deformation.

### 2.7. Statistics

Data are presented as mean ± standard deviation (SD). The Shapiro–Wilk test was used to assess the normality of data distribution. Differences between independent groups were initially evaluated using the non-parametric Mann–Whitney U test, with statistical significance at *p* ≤ 0.05.

To account for the repeated measurements obtained from individual donors across multiple shear rates, linear mixed-effects modeling was additionally applied. In this model, donor identity was treated as a random effect, while shear rate and group (CLL vs. healthy donors) were treated as fixed effects. The interaction between group and shear rate was also included to evaluate potential differences in deformation responses between groups across flow conditions. For each software-based analysis (LabVIEW and Python), F-values, *p*-values, and partial eta squared (η^2^_p_) were calculated for the fixed effects. F-statistics assess the effect of fixed factors relative to residual variability, with degrees of freedom (df) approximated using the Satterthwaite method. Statistical significance was evaluated using *p*-values, and the magnitude of effects was quantified using partial eta squared (η^2^_p_), where higher values indicate a larger proportion of variance explained by the factor.

Agreement between deformation index (DI) values obtained from the two image-analysis pipelines was evaluated using Pearson correlation analysis and Bland–Altman analysis, which provided estimates of mean bias and 95% limits of agreement.

Statistical analyses were performed using OriginPro 2018 (OriginLab Corporation, Northampton, MA, USA) and JASP (Version 0.18; JASP Team, University of Amsterdam, Amsterdam, The Netherlands). Linear mixed-effects modeling was conducted in JASP.

## 3. Results

### 3.1. Clinical and Hematological Characteristics of the CLL Patients and Healthy Individuals

Nine patients with CLL, five of whom were men, were enrolled in this study. Seven patients were classified as having early-stage disease (Rai stage 0). These individuals were asymptomatic or presented only mild clinical manifestations that did not require therapeutic intervention. It should also be noted that two patients in this group were newly diagnosed.

The clinical and hematological characteristics of the studied groups are summarized in Table 1. The control and CLL groups were comparable in age and sex distribution.

RBC parameters remained within the reference ranges in both groups, suggesting the absence of overt anemia in untreated CLL patients. However, a modest increase in red cell distribution width (RDW), together with substantial variability, suggests subtle heterogeneity in erythrocyte size. It is worth noting that RDW values above the reference range were observed in three patients.

Pronounced and statistically significant elevations in white blood cell (WBC) and absolute lymphocyte counts were observed in CLL patients compared with healthy controls (*p* < 0.05). The wide range of values observed in the untreated CLL group indicates substantial interpatient heterogeneity, characteristic of the disease and reflecting variable levels of lymphocytic expansion.

### 3.2. Development of a Software Application for the Analysis of Red Blood Cell Deformation Under Flow Conditions

**Figure 1:**
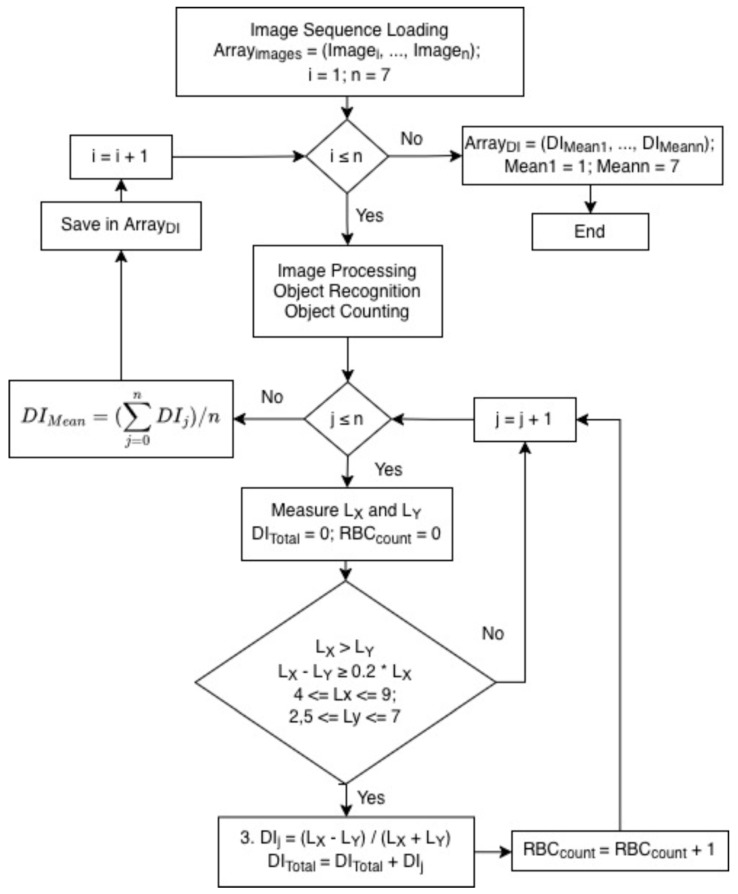
Block diagram of the algorithm for the analysis of RBC deformation. The workflow illustrates the main steps: image acquisition from the BioFlux microfluidic system, detection and segmentation of individual RBCs, ellipse fitting to determine the cells, counting of cells, defining cell axes and calculation of the deformability index (DI) for each cell.

To quantitatively assess RBC deformability, a dedicated algorithm was developed to analyze images of cells under dynamic flow. The overall workflow of the algorithm is summarized in a block diagram (Figure 1), highlighting the sequence of image acquisition, preprocessing, cell detection, and deformability index calculation.

The algorithm was implemented in two platforms, i.e., LabVIEW (commercial) and Python (open-source), to allow cross-validation of results.

**Figure 2:**
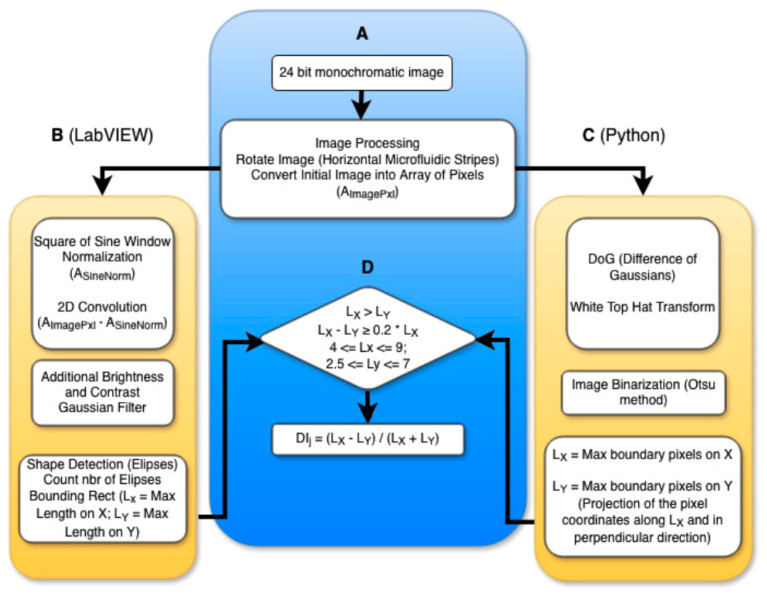
Schematic comparison of the RBC image-analysis workflows implemented in LabVIEW and Python. (**A**) Pre-processing step common to both methods: the original microfluidic image is rotated to align the streamlines horizontally along the X axis. (**B**) Image-processing pipeline used in the LabVIEW implementation, including normalization of the square of a sine-window kernel, 2D convolution filtering of the original image with the horizontal sine wave kernel, brightness and contrast adjustment, Gaussian filtering, and object detection. The recognition and counting of the cells is realized using an ellipse fitting technique. Cell dimensions (L_X_ and L_Y_) are extracted from the bounding rectangle enclosing each cell contour. (**C**) Image-processing pipeline used in the Python implementation, involving the creation of two versions of the same monochrome image with different thresholds of the applied Gaussian filter, Difference of Gaussians (DoG) filtering, top-hat transformation, and segmentation using the Otsu thresholding method. The diameters of each cell L_X_ and L_Y_ were determined. The two outermost X coordinates of the pixels that are part of the cell defined the orientation and size of L_X_. All pixels of the cell are then projected onto this axis and onto the perpendicular direction. The extrema of these projections define the dimensions of the smallest enclosing rectangle aligned with Lx. (**D**) Common post-processing and cell-selection criteria, applied in both approaches for RBC identification and calculation of the deformation index (DI).

Digital images of RBCs were acquired using the BioFlux microfluidic system (Figure 2A). For each donor, seven 24-bit RBC monochrome images (ArrayImages = {Image_1_, …, Image_7_}) were captured, each corresponding to a progressively increasing shear rate.

RBC images were analyzed using two software pipelines, implemented in LabVIEW and Python, with a common initial preprocessing step in which the original microfluidic image was rotated to align the streamlines horizontally along the X axis. In the LabVIEW workflow, images were smoothed by 2D convolution filtering using a square sine-window kernel, brightness, contrast adjustment, and Gaussian filtering. Objects were detected based on ellipse approximation, and the cell dimensions L_X_ and L_Y_ were obtained from the bounding rectangle of each object with sides corresponding to the major and minor diameters of the ellipse, respectively. In the Python workflow, preprocessing included creation of two versions of the original image with Gaussian smoothing with different thresholds, Difference of Gaussians filtering, a horizontal top-hat transformation, and segmentation using the Otsu thresholding method. For each detected object, L_X_ and L_Y_ (diameters of each cell) were determined. The two outermost X coordinates of the pixels that (part of the cell) defined the orientation and size of L_X_. All pixels of the cell are then projected onto this axis and onto the perpendicular direction. The extrema of these projections define the dimensions of the smallest enclosing rectangle aligned with L_X_. The length along L_X_ is denoted as L_X_, and the length along the perpendicular direction is denoted as L_Y_.

This workflow ensures that, despite differences in preprocessing and segmentation strategies, the geometric measurements and DI calculation are consistent across both implementations.

#### 3.2.1. Software Application Using LabVIEW

The background of the 24-bit RGB monochrome input noisy image consists mainly of microfluidic streamlines, which are characterized by a smooth variation in the horizontal direction and sharp boundaries in the vertical direction. To suppress the microfluidic streamlines and to highlight the RBC, 2D convolution with two input arrays is applied during image processing. One of the inputs contains one of the 8-bit channels of the 24-bit monochrome initial RGB image (the pixel values of the red, green, and blue 8-bit channels are equal, so the selection did not affect the result). A sinusoidal function is used for creation of the second input array (convolution kernel), producing a symmetric window with maximum intensity at the center, decreasing toward the edges. The values of the positive half cycle of the sine wave function (sine wave window) are squared and then normalized by dividing each element by the sum of all values, ensuring that the total sum equals one. This normalization preserves the relative intensity of the central pixel while maintaining overall image brightness.

2D Convolution of the pixel array with the sine window blurred the background while preserving the RBCs contrast. Subtracting the filtered image from the original removed the background, leaving RBCs clearly visible for analysis (Figure 2B). Contrast was further enhanced to facilitate object detection. This approach effectively reduces background noise in a manner comparable to a Gaussian distribution, enabling reliable detection and quantitative evaluation of RBC deformation.

Following background suppression, individual ellipsoidal objects (diameter 3–50 pixels) were detected and segmented, and their X–Y coordinates were recorded. An ellipse was fitted to each object, and the lengths of the major (L_X_) and minor (L_Y_) diameters were measured, corresponding to the maximal extents along the X (flow) and Y (transverse) axes (Figure 2B). Objects were classified as deformed RBCs only if they satisfied the following criteria (Figure 2D):

(a) L_X_ > L_Y_
(b) L_X_ – L_Y_ ≥ 0.2 × L_X_
(c) L_X_ and L_Y_ within the ranges 4 ≤ L_x_ ≤ 9 and 2.5 ≤ L_Y_ ≤ 7 pixels

For each accepted RBC, the deformability index (DI) was calculated as in Equation (1).

All DI values within an image were summed to obtain the total deformability index (DI_Total_), and the mean deformability index (DI_Mean_) was computed for each image. DI_Mean_ values from all seven images were stored for each donor.

#### 3.2.2. Implementation of the Sine Window Filter

Mathematical functions, such as sine window functions, are widely used in digital filter design. Mathematically, when another function is multiplied by a window function, the product is also zero-valued outside the interval. In LabVIEW, the sine window was generated using the Sine Pattern.vi module. A sine window represents half a cycle of a sinusoidal function. The number of samples was selected to optimize signal smoothing. The sine window values were squared and normalized by dividing each element by the sum of all kernel elements to preserve overall image intensity

In the performed image processing, a 24-bit RGB monochrome noisy image was converted to an array of pixel values. In particular, all three channels of the monochrome image (Red, Blue, and Green 8-bit channels) have equal pixel values.

In LabVIEW 2D convolution instrument used two input arrays. The first input array contained the pixel intensity values of one of the three 8-bit channels (Red, Blue, or Green channel). The second input array consisted of the normalized sine window function values, known as a convolution kernel. Because the background structures in the microfluidic images (flow streamlines) vary smoothly along the horizontal direction, a one-dimensional convolution kernel with 100 elements was applied along the X axis. The kernel values correspond to 100 samples of a half-cycle sine function that are squared and normalized to preserve the overall image intensity. The filtered output array retained the same dimensions as the first array in 2D convolution filtering. As a result, the image after 2D convolution suppresses the microfluidic flow jets to highlight the RBC as bright objects.

#### 3.2.3. Software Application Using Python Programming Language

First, the 24-bit grayscale image was smoothed using two Difference Gaussians (DoG) filters, implemented by subtracting two Gaussian-smoothed versions of the image with different standard deviations σ_1_ = 0.7 and σ_2_ = 16). This band-pass filtering step suppressed slowly varying background illumination while enhancing localized high-frequency structures corresponding to individual cells. As a result, the cells appeared as distinct bright regions relative to the background.

Next, a top-hat transformation was applied using a linear structuring element of length 45 pixels, removing elongated background horizontal elements (e.g., flow streaks) while preserving small, compact objects such as individual cells. The resulting image was then binarized using the Otsu thresholding method, multiplied by a factor of 1.06 to optimize segmentation. The boundary objects were removed, and only objects with areas between 50 and 130 pixels were retained (Figure 2C).

An additional criterion was applied to exclude artifacts: the deformation index (DI) ≤ 0.35.

After meeting all requirements mentioned above, the objects are classified as RBCs.

The two most distant points of the segmented cell define the primary axis L_X_, determining its orientation and maximum extent (see Section 2.6). Projecting all pixels onto this axis and its perpendicular, the extrema define the smallest enclosing rectangle, with lengths along the axes denoted L_X_ and L_Y_.

For each detected cell, L_X_ and L_Y_ were measured, and the deformability index was then calculated using the same formula as in the LabVIEW analysis (Figure 2D). The DI values were then averaged for each image (i.e., for each shear rate) and used for further statistical analysis.

### 3.3. Limitations and Sources of Inter-Platform Variability

Although the analytical workflow, DI definition, and object selection criteria were harmonized across the LABVIEW and Python implementations, systematic differences in absolute DI values were observed between software platforms. These discrepancies are primarily attributable to platform-specific differences in image processing implementation, including numerical precision, convolution and interpolation procedures, thresholding strategies, and object segmentation algorithms. Minor variations in background suppression, kernel normalization, and morphological filtering can lead to differences in estimated cell boundaries, particularly for weakly deformed or partially overlapping cells, thereby affecting the calculated coordinates (L_x_ and L_y_) and the resulting DI values.

In addition, default parameter handling, rounding behavior, and internal optimization routines differ among software environments, which may further contribute to small but consistent offsets in DI measurements. Importantly, despite these differences in absolute values, all platforms demonstrated comparable relative trends and consistently distinguished between healthy donors and patients. Therefore, the observed inter-platform variability should be interpreted as a methodological limitation inherent to cross-software image analysis rather than as a limitation of the deformability metric itself.

### 3.4. Comparison Between Deformability Index Values for Healthy Individuals and Patients with CLL

The obtained data were further analyzed to characterize shear-dependent RBC deformability in healthy controls and untreated CLL patients. The number of RBCs analyzed per donor and shear rate is summarized in Supplementary Table S1, reflecting the throughput of the high-resolution imaging approach used in this study.

Cell deformation was assessed across a shear rate range of 89–625 s^−1^, and RBC deformability was expressed as the Deformability Index (DI) (Table 2). In both groups, DI generally increased with rising shear stress, indicating a shear-dependent enhancement of RBC deformability. This behavior is consistent with normal erythrocyte biomechanics, as RBCs are expected to deform more under higher shear stress. Differences between RBCs from healthy controls and patients with CLL are illustrated in Supplementary Figure S2, which presents representative images obtained under intermediate flow conditions, demonstrating characteristic cell deformation within the microfluidic channel.

#### 3.4.1. Python-Based Analysis

**Figure 3:**
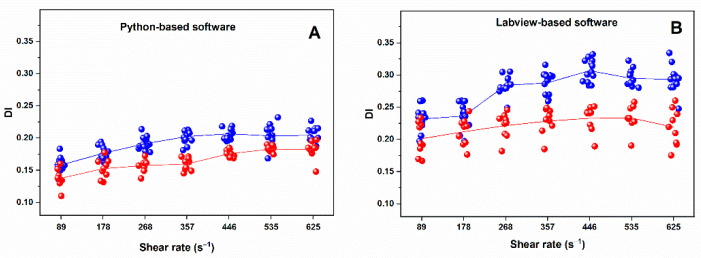
(**A**) Dependence of deformation index (DI) on shear rate for RBCs isolated from healthy individuals (blue circles) and untreated CLL patients (red circles) obtained using Python-based analysis; (**B**) Dependence of deformation index on shear rate for RBCs isolated from healthy individuals (blue circles) and untreated CLL patients (red circles) obtained using LabVIEW-based software. Individual points represent measurements from each donor, illustrating the variability within the group at each shear rate, while lines connect the mean values for each group at the corresponding shear stress levels.

Analysis using the Python-based software demonstrated across most of the shear rate range (89–625 s^−1^), DI values were lower in untreated CLL patients compared with healthy controls (Table 2, Figure 3). Statistically significant differences (*p* < 0.05, Mann–Whitney U test) were observed at intermediate shear rate levels corresponding to 268, 357, and 446 s^−1^, whereas differences at the lowest and highest shear rates did not reach statistical significance. The largest absolute group differences were observed at intermediate to high shear rates (268, 357, and 446 s^−1^), suggesting a pronounced impairment of RBC deformability in CLL under these conditions.

In healthy individuals, DI increased steadily up to 446 s^−1^; beyond this point, it reached a plateau and showed a subtle decline at 535 and 625 s^−1^. In contrast, RBCs from CLL patients showed a markedly attenuated (blunted) shear-dependent response. After an initial increase between 89 and 178 s^−1^, DI remained relatively unchanged between 178 and 357 s^−1^, followed by a modest rise at 446 s^−1^, after which further increases in shear stress resulted in only minimal changes in DI.

Overall, these findings demonstrate a systematic and significant reduction in RBC deformability in untreated CLL patients and a blunted shear-dependent deformability response compared with healthy controls.

#### 3.4.2. LabVIEW-Based Analysis

Analysis using the LabVIEW-based software revealed a pattern of RBC deformability broadly consistent with the Python-based results, although absolute DI values were systematically higher across all shear rates (Table 2, Figure 3). In healthy controls, LabVIEW-derived DI increased progressively from 0.236 ± 0.029 at 89 s^−1^ to a peak of 0.307 ± 0.016 at 446 s^−1^, followed by a slight decrease at higher shear rates (0.291 ± 0.025 at 535 s^−1^ and 0.293 ± 0.024 at 625 s^−1^).

In untreated CLL patients, DI values calculated with LabVIEW were consistently lower than those of healthy controls at all shear rates. The increase in DI with rising shear stress was attenuated, reflecting a blunted shear-dependent response. DI rose gradually between 89 s^−1^ and 446 s^−1^, followed by a modest increase to 0.254 ± 0.018 at 625 s^−1^ (Table 2, Figure 3). Compared with healthy RBCs, CLL RBCs exhibited a marked reduction in deformability at intermediate shear rates (178–446 s^−1^), where differences reached statistical significance (*p* ≤ 0.05), with the most robust differences observed at 357–446 s^−1^.

Despite differences in the absolute magnitude of DI values between the two analytical pipelines, both methods demonstrated the same biological trend of reduced RBC deformability in CLL patients.

#### 3.4.3. Mixed-Effects Statistical Analysis

To account for the repeated-measures structure of the dataset, in which each donor contributed measurements across multiple shear-rate conditions, RBC deformability was further analyzed using a linear mixed-effects model. In this model, donor identity was treated as a random effect, while group (CLL vs. healthy donors) and shear rate were included as fixed effects. This approach accounts for correlations between repeated measurements obtained from the same donor across different shear conditions.

The mixed-effects analysis revealed a highly significant group effect for both analytical pipelines, confirming that RBCs from untreated CLL patients exhibit markedly reduced deformability compared with healthy donors across the tested shear range (Table 3). For the LabVIEW-derived data, the group effect was particularly pronounced (F = 93.56, *p* < 0.001, η^2^_p_ = 0.82), indicating a very large effect size. A similarly strong group effect was observed for the Python-derived data (F = 61.72, *p* < 0.001, η^2^_p_ = 0.76).

A significant main effect of shear rate was also detected in both analyses (LabVIEW: F = 35.22, *p* < 0.001, η^2^_p_ = 0.64; Python: F = 49.38, *p* < 0.001, η^2^_p_ = 0.71), reflecting the expected shear-dependent increase in RBC deformability. RBCs from untreated CLL patients exhibited a reduced shear-dependent increase in DI, indicating a blunted mechanical response to rising shear stress.

A significant group × shear rate interaction was identified in both analytical approaches (LabVIEW: F = 8.49, *p* < 0.001, η^2^_p_ = 0.30; Python: F = 2.86, *p* = 0.012, η^2^_p_ = 0.13). This interaction indicates that the magnitude of the shear-dependent deformability increase differs between groups. Specifically, CLL RBCs showed smaller increases in DI across the shear-rate range compared with healthy controls.

Overall, the mixed-effects modeling confirms that CLL is associated with a significant reduction in RBC deformability and an altered shear-response profile, findings that are consistently detected using both the Python-based and LabVIEW-based analysis pipelines. Despite differences in absolute DI values between LabVIEW and Python pipelines, both analytical approaches reveal consistent biological trends, supporting the robustness of the observed impairment in RBC mechanical properties in untreated CLL.

**Figure 4:**
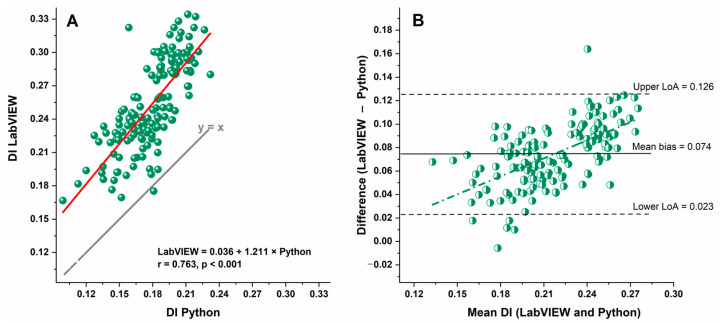
Comparison of deformability index (DI) values obtained using LabVIEW and Python pipelines. (**A**) Correlation between DI values, showing a strong positive association (r = 0.763, *p* < 0.001); red line shows linear regression fit, indicating a slight systematic deviation from the identity line (y = x). (**B**) Bland–Altman plot showing mean bias (0.074, solid line) and 95% limits of agreement (0.023–0.126, dashed lines). Most measurements fall within the limits, with a proportional bias at higher DI values likely due to differences in preprocessing and segmentation strategies, including background subtraction and thresholding.

Agreement between the LabVIEW and Python pipelines was further evaluated using Bland–Altman analysis. The mean bias between the two methods was 0.074, with 95% limits of agreement ranging from 0.023 to 0.126 (Figure 4). Approximately 97% of observations fell within the limits of agreement (0.023 to 0.126), indicating good concordance between the two approaches. Linear regression of the differences against the mean DI values revealed no significant proportional bias, suggesting that the magnitude of disagreement did not depend on the level of deformation.

Figure 4A compares deformability index (DI) values obtained using the LabVIEW and Python pipelines. DI values showed a strong positive correlation (r = 0.763, *p* < 0.001), though the regression line deviated from the identity line (y = x), indicating a systematic difference. The Bland–Altman analysis (Figure 4B) revealed a mean bias of 0.074, with 95% limits of agreement from 0.023 to 0.126, showing most measurements were consistent between pipelines. A proportional bias was observed at higher DI values, likely reflecting differences in preprocessing and segmentation strategies, such as background subtraction and thresholding, which can affect contour detection for highly deformed cells.

#### 3.4.4. Comparison Between Python- and LabVIEW-Based Calculations

In line with the above observations, DI values obtained using LabVIEW-based software were consistently slightly higher than those calculated with the Python-based platform across all shear rates (Table 2, Figure 3). This systematic upward shift in absolute values likely reflects differences in image-processing implementation, filtering strategies, and numerical handling between the two computational environments.

Despite this upward shift in absolute values, both analytical approaches exhibited the same qualitative behavior: (i) an progressive increase in DI with rising shear rate in healthy controls, reaching a plateau at higher shear levels; (ii) lower DI values in untreated CLL patients compared with healthy controls across the measured shear range; and (iii) a diminished (blunted) shear-dependent deformability response in CLL RBCs compared with controls, particularly evident at intermediate shear rates. The most pronounced and statistically significant differences between groups were observed at intermediate shear rates (268–446 s^−1^), suggesting that these flow conditions may be particularly sensitive for detecting subtle mechanical alterations in RBCs associated with CLL.

Pearson correlation analysis demonstrated a strong positive correlation between the two software platforms. The correlation coefficient was very high in the healthy control group (r = 0.96) and slightly lower in the CLL group (r = 0.78), indicating substantial agreement between platforms in both cohorts. The somewhat reduced correlation in the patient group likely reflects greater biological variability and dispersion of DI values among CLL samples rather than methodological inconsistency.

These findings indicate that although the two computational approaches yield different absolute DI values, they preserve consistent relative trends and intergroup differences. This concordance reinforces the robustness and reproducibility of the observed impairment in RBC deformability in untreated CLL patients, independent of the software platform used for analysis.

## 4. Discussion

In this study, RBC deformability, determined under controlled flow conditions, was studied using a microfluidic imaging approach combined with harmonized image-analysis pipelines implemented in two software platforms. Deformability was quantified using a geometrically defined DI, enabling both biological comparison between healthy donors and CLL patients and technical comparison across software environments.

### 4.1. RBC Deformability in Healthy Donors

In healthy donors, DI increased progressively with rising shear rate and approached a plateau at higher shear levels, reflecting the expected biomechanical adaptation of RBCs to hydrodynamic stress. This trend was observed in all software platforms, despite minor differences in absolute DI values. The gradual increase in DI likely reflects shear-induced alignment and elongation of RBCs as they adapt to sustained flow conditions within the microfluidic channel. This response is consistent with the viscoelastic behavior of the RBC membrane–cytoskeletal complex. Under physiological conditions, the membrane skeleton permits RBCs to undergo marked reversible deformation, reflecting their intrinsic mechanical adaptability under external loading [27]. Such behavior is consistent with established hemorheological principles, whereby healthy RBCs demonstrate enhanced deformability in response to increasing shear rates, facilitating efficient microvascular transit [28].

The progressive increase in DI with increasing shear rate further supports the physiological relevance of the microfluidic model and its ability to capture flow-dependent RBC behavior under controlled conditions. In addition, the consistent response observed across analysis platforms demonstrates that the proposed image-analysis framework is sufficiently sensitive to detect subtle mechanical variations. The preservation of this shear-dependent trend indicates that the deformability index reliably reflects relative changes in cell deformation despite minor implementation-related differences in absolute values.

### 4.2. Altered Deformability Patterns in CLL Patients

In contrast to those of healthy donors, RBCs from CLL patients exhibited a reduced slope of DI increase, with irregular and non-monotonic patterns across successive shear conditions. The absence of a consistent trend and the attenuated response indicate heterogeneous or impaired mechanical adaptability to flow. Several pathophysiological mechanisms likely contribute to this altered behavior.

These changes may reflect pathological alterations in RBC membrane properties, cytoplasmic viscosity, or interactions with plasma components characteristic of CLL. The disease is associated with systemic inflammation and increased oxidative stress, detectable even at early stages. For example, Yan et al. reported broadly elevated serum cytokine levels in CLL patients compared with healthy individuals, with some cytokines linked to core disease-associated pathways and others correlating with aggressive disease features [29]. Rozovski et al. further demonstrated that inflammatory cytokines both originate from and reinforce pro-inflammatory signaling within the malignant clone, while interactions with immune cells in the microenvironment sustain a chronic inflammatory state [30]. These findings indicate that persistent inflammatory signaling is a fundamental feature of CLL and contributes to systemic oxidative imbalance. Chronic inflammatory signaling and oxidative stress in CLL may therefore extend beyond the malignant clone and influence circulating RBCs.

Oxidative modifications of membrane lipids and cytoskeletal proteins, such as spectrin and ankyrin, can increase membrane stiffness and reduce elastic resilience, ultimately impairing the cell’s ability to adapt to shear stress. Supporting this mechanism, our recent work demonstrated pronounced nanomechanical alterations in RBCs from CLL patients, indicative of changes in membrane structural integrity and mechanical responsiveness [31]. These nanoscale mechanical changes provide a plausible explanation for the reduced and blunted deformability response observed in the present microfluidic analysis. A quantitative relationship between membrane stiffness and functional deformability was established by correlating Young’s modulus (E), measured in [28], with the deformability index (DI) at a defined shear rate (e.g., 357 s^−1^). As expected, stiffer RBCs (higher E) exhibited lower DI values, with a strong negative correlation (Pearson r = −0.96), supporting the link between nanomechanical alterations and impaired shear-dependent deformability in CLL.

The observed DI fluctuations in CLL patient samples are consistent with impaired rheological regulation and may contribute to microcirculatory dysfunction and elevated vascular resistance, as reduced RBC deformability is known to hinder capillary flow and increase hemodynamic burden in the microvasculature [32].

An important finding of our study is that the largest DI differences between healthy and CLL RBCs occurred at intermediate shear rates. At low shear rates, the absence of a statistically significant difference in DI between control and CLL groups may reflect an insufficient amount of deformation forces to fully reveal the underlying mechanical deficits in CLL RBCs. Under these conditions, even cells with impaired membrane or cytoskeletal integrity can maintain near-normal or comparable to healthy state shape, thereby masking subtle defects in deformability. This phenomenon is strongly influenced by the level of applied shear stress and by cellular mechanical heterogeneity. As discussed by Nermi L. Parrow et al., deformability measurements are strongly dependent on applied shear stress, and low-stress conditions may underestimate mechanical heterogeneity within RBC populations [33]. Similarly, Yujin Heo et al. demonstrated that erythrocyte deformability profiles vary markedly with shear stress, with mechanical differences becoming more evident only under sufficiently high deformation forces [34].

In contrast, intermediate shear levels generate adequate mechanical stress to expose heterogeneity in mechanical responses, highlighting the reduced elasticity and adaptability of CLL RBCs. At very high shear rates, RBC elongation approaches a mechanical limit, reducing the relative contrast between the two groups. This interpretation is consistent with the parameterization of elongation index–shear stress curves described by Baskurt et al., showing that RBC elongation progressively approaches a maximal value (EI_max_) at high shear stress, where additional increases in shear result in only minimal changes in deformation [35]. Intermediate shear conditions, therefore, appear to represent a dynamic range in which membrane–cytoskeleton coupling and viscoelastic adaptation are most critical, thereby unmasking subtle impairments in CLL RBCs.

It should be noted that these altered deformability patterns were consistently detected across both analytical platforms. This reproducibility strengthens the conclusion that the observed mechanical alterations are biological in origin rather than methodological artifacts.

Reduced and mechanically inconsistent RBC deformability may impair microvascular perfusion and tissue oxygen delivery, particularly under conditions requiring dynamic adaptation to variable flow. Although anemia is not typical in early-stage CLL, subclinical rheological alterations may still contribute to microcirculatory dysfunction. Thus, microfluidic deformability assessment may provide additional insight into disease-associated systemic alterations beyond conventional hematological parameters.

Collectively, these findings indicate that untreated CLL is associated with measurable impairment in RBC mechanical adaptability under shear stress, characterized not only by reduced deformability but also by an altered shear-response pattern. This altered biomechanical signature may represent a subtle but clinically relevant manifestation of systemic disease activity.

### 4.3. Cross-Platform Comparison and Methodological Implications

A main aim of this study was to assess the impact of software platform selection on deformability quantification. Similar microchannel-based approaches have previously been used to investigate RBC mechanics and flow-induced deformation, highlighting the complex interplay between cell mechanical properties, channel geometry, and hydrodynamic forces [36]. While LABVIEW consistently yielded higher absolute DI values compared to Python, both platforms demonstrated concordant relative trends and preserved discrimination between healthy donors and CLL patients. This finding indicates that absolute DI values may depend on the specific image-processing pipeline, whereas relative changes and group-level patterns remain robust and biologically informative.

The observed quantitative differences are primarily attributable to differences in preprocessing, filtering, and segmentation strategies implemented in the two pipelines. The LabVIEW workflow relies on sinusoidal window smoothing combined with Gaussian filtering and brightness/contrast adjustments, whereas the Python-based pipeline employs Difference-of-Gaussians (DoG) filtering followed by a top-hat transformation to enhance compact objects and suppress elongated background structures such as microfluidic streamlines. These distinct filtering strategies influence the delineation of cell boundaries and, therefore, the detected object contours.

Because the deformation index is calculated from the major and minor axes of the detected cell shape, even small variations in the segmentation of the cell boundaries can propagate into measurable differences in L_X_ and L_Y_, and consequently in DI. Thus, the discrepancies observed between the two implementations likely reflect methodological differences in preprocessing and segmentation rather than differences inherent to the software platforms themselves.

These results emphasize the importance of clearly documenting image-processing pipelines and harmonized analytical criteria, particularly when comparing deformability measurements obtained across different software implementations or laboratories.

### 4.4. Interpretation of the Deformation Index

In the present study, the deformation index (DI) should be interpreted as an effective measure of RBC deformability that reflects not only intrinsic membrane mechanical properties but also the combined influence of cytoskeletal integrity, cell geometry, and the hydrodynamic conditions encountered in the microfluidic channel. Microfluidic approaches commonly quantify deformability by the elongation of single cells under flow, yielding a deformation index widely used as a biomechanical indicator in microcapillary and constriction-based assays [37]. Related high-throughput approaches include real-time deformability cytometry (RT-DC), introduced by Otto et al., which enables rapid mechanical phenotyping of thousands of cells per second based on hydrodynamic deformation in microfluidic channels, allowing mechanical characterization of thousands of cells per second [12]. These techniques demonstrate the growing role of label-free mechanical phenotyping in cell characterization and complement microfluidic imaging approaches such as the one employed in the present study.

At low deformation rates, interactions with channel walls and residual adhesion forces can influence the measured DI, and under these conditions, we did not observe statistically significant differences between healthy and patient RBCs. This highlights that single-cell microfluidic deformability assays capture context-dependent factors related to cell–surface interactions and flow confinement, in addition to membrane mechanics. In contrast, established techniques such as laser diffraction ektacytometry measure the ensemble deformability of RBC populations under highly controlled shear stress in a viscous medium, providing complementary insights into cell deformability but lacking single-cell resolution [38]. While ektacytometry remains a clinical standard for assessing bulk deformability, single-cell microfluidic assays offer the advantage of resolving morphological deformation under physiologically relevant constraints, albeit with additional factors that can modulate the DI. Taken together, these methods provide a broader perspective on RBC mechanical behavior and underscore the need to consider both intrinsic and context-dependent contributions when interpreting deformability metrics.

### 4.5. Clinical and Methodological Relevance

The ability to distinguish healthy and pathological deformability patterns using a microfluidic DI-based approach highlights the potential of this methodology for clinical applications. Although this study is not intended for diagnostic purposes, the observed alterations in deformability in CLL patients suggest that RBC mechanics may serve as a complementary indicator reflecting disease-related systemic effects. Moreover, the demonstrated sensitivity of DI to both biological conditions and analytical implementation highlights the need for standardized deformability metrics if such approaches are to be integrated into clinical studies.

### 4.6. Study Limitations

Finally, it should be noted that the present microfluidic assay evaluates RBC deformation under confined flow conditions and therefore reflects a combination of intrinsic mechanical properties and experimental geometry. Consequently, DI values should be interpreted primarily in a comparative rather than absolute mechanical sense.

## 5. Conclusions

In this study, a microfluidic image flow approach was applied to quantify RBC deformability and to compare healthy individuals with untreated CLL patients. The results demonstrated reduced deformability and a blunted shear-dependent response of RBCs in CLL, particularly at intermediate shear rates, indicating altered biomechanical behavior under flow conditions.

Implementation of the analysis algorithm in both LabVIEW and Python showed that, despite systematic differences in absolute DI values, both developed software applications preserved consistent relative trends and group discrimination. This confirms that the observed deformability alterations are robust and biologically relevant, while highlighting the influence of image-processing implementation on absolute measurements.

Overall, the presented workflow combines microfluidic experimentation with automated image analysis to provide a reproducible method for assessing RBC mechanics. Further standardization of analysis pipelines and validation in larger cohorts may support broader application of this approach for studying hemorheological alterations in different diseases.

## Figures and Tables

**Table 1:** Clinical (age, gender, and Rai stage) and hematological indices (RBC count; hemoglobin, Hb; hematocrit, Ht; mean corpuscular volume, MCV; mean corpuscular hemoglobin, MCH; mean corpuscular hemoglobin concentration, MCHC; red blood cell distribution width, RDW; white blood cell count, WBC and lymphocyte count) determined for healthy controls and CLL patients. Data are presented as mean ± standard deviation (SD).

Parameters	Reference Value	Studied Groups	
		Healthy Controls (*n* = 13)	Untreated CLL Patients (*n* = 9)
Age (years)	-	58.00 ± 7.4	63.89 ± 12.01
Gender (F/M)		8/5	4/5
Rai stage			0–1
RBC count (T/L)	4.60–6.20	4.97 ± 0.23	4.98 ± 0.39
Hb (g/L)	140.00–180.00	161.40 ± 8.09	146.44± 11.86
Ht (L/L)	0.40–0.54	0.48 ± 0.01	0.44 ± 0.03
MCV (fl)	80.00–95.00	89.10 ± 3.74	87.93 ± 5.01
MCH (pg/L)	27.00–32.00	30.55 ± 1.33	29.43 ± 1.69
MCHC (g/L)	320.00–360.00	344.75 ± 4.15	335.00 ± 8.62
RDW %	11.60–14.80	13.88 ± 0.80	14.50 ± 1.32
WBC	3.50–10.50	6.3 ± 1.1	11.7 ÷ 157.5 *
Lymphocytes (ABS)	1.10–3.80	1.91 ± 0.17	6.70 ÷ 144.28 *

* Indicates statistically significant difference (*p* < 0.05) in the values of laboratory indices for CLL patient groups compared with the control values.

**Table 2:** Deformability Index (DI) of RBCs from healthy individuals and untreated CLL patients at different shear rates, calculated using Python-based software and LabVIEW-based software. Data are presented as mean ± standard deviation. * Indicates a statistically significant difference at *p* < 0.05 according to the Mann–Whitney U test.

Shear Rate (s^−1^)	Python-Based Software		LabVIEW-Based Software	
	Healthy Controls	Untreated CLL Patients	Healthy Controls	Untreated CLL Patients
89	0.157 ± 0.011	0.136 ± 0.014	0.236 ± 0.029	0.201 ± 0.023
178	0.176 ± 0.011	0.151 ± 0.014	0.271 ± 0.022	0.212 ± 0.020 *
268	0.191 ± 0.010	0.156 ± 0.009 *	0.284 ± 0.020	0.221 ± 0.018 *
357	0.200 ± 0.014	0.159 ± 0.008 *	0.293 ± 0.017	0.228 ± 0.019 *
446	0.205 ± 0.010	0.175 ± 0.007 *	0.307 ± 0.016	0.233 ± 0.019 *
535	0.202 ± 0.018	0.181 ± 0.005	0.291 ± 0.025	0.233 ± 0.020 *
625	0.204 ± 0.013	0.182 ± 0.014	0.293 ± 0.024	0.219 ± 0.029 *

**Table 3:** Results of the linear mixed-effects model analysis evaluating the effects of group (CLL vs. healthy donors), shear rate, and their interaction on the deformation index (DI). Donor identity was included as a random effect to account for repeated measurements across shear rates. Group and shear rate were treated as fixed effects. For each software-based analysis (LabVIEW and Python), F-values, *p*-values, and partial eta squared (η^2^_p_) are reported for the fixed effects.

Software-Based Analysis	Factor	df	F	*p*	η^2^_p_
LabVIEW	Group	1, 20	93.56	<0.001	0.82
	Shear rate	6, 120	35.22	<0.001	0.64
	Group × Shear rate	6, 120	8.488	<0.001	0.30
Python	Group	1, 20.1	61.72	<0.001	0.76
	Shear rate	6, 119.2	49.38	<0.001	0.71
	Group × Shear rate	6, 119.2	2.86	0.012	0.13

## Data Availability

All data are contained within the manuscript and available upon request.

## Supplementary Materials

The following supporting information can be downloaded at: https://www.mdpi.com/article/10.3390/mi17030389/s1, Figure S1: Schematic of the microfluidic system used in this study. The system integrates a BioFlux 200 flow control pump, LumaScope 620 inverted fluorescence microscope with camera, BioFlux 24-well plates (350 × 75 μm^2^ channels, 0–20 dyn/cm^2^), a mini-incubator (37 °C, 5% CO_2_), and PC-based control/analysis software. Representative images of deformed RBCs from healthy controls (A,C) and patients with CLL (B,D), analyzed using LabVIEW-based software (A,B) and Python-based software (C,D) under representative flow conditions (268 s^−1^). The images illustrate the characteristic deformation of RBCs within the microfluidic channel. Scale bar—10 µm. Table S1. Number of analyzed RBCs per shear rate in Healthy controls and CLL samples.
