# Supplementary material for: Comparative Evaluation of Two Image-Analysis Software Platforms for Microfluidic Assessment of Red Blood Cell Deformability in Chronic Lymphocytic Leukemia
Source: Micromachines (Basel). 2026 Mar 23;17(3):389. doi: 10.3390/mi17030389 (PMC13028752; doi:10.3390/mi17030389)
Supplement: Supplementary file 1 [file micromachines-17-00389-s001.zip › micromachines-4184346-supplementary.pdf]

Comparative Evaluation of Two Image-Analysis Software Platforms for Microfluidic Assessment of Red Blood Cell Deformability in Chronic Lymphocytic Leukemia

Anika Alexandrova-Watanabe [1,2,†], Tihomir Tiankov [1,2,†], Aleksandar Iliev [1,2], Ariana Langari [2,3], Miroslava Ivanova [2,3], Lidia Gartcheva [4], Margarita Guenova [4], Emilia Abadjieva [1,2], Sashka Krumova [3] and Svetla Todinova [2,3,*]

[1] Institute of Mechanics, Bulgarian Academy of Sciences, "Acad. G. Bontchev" Str. 4, 1113 Sofia, Bulgaria; anikaalexandrova@abv.bg (A.A.-W.); tiho_bg@abv.bg (T.T.); alxndr.iliev@gmail.com (A.I.); abadjieva@gmail.com (E.A.)
[2] Center of Competence for Mechatronics and Clean Technologies "Mechatronics, Innovation, Robotics, Automation and Clean Technologies"—MIRACle, "Acad. G. Bontchev" Str. 4, 1113 Sofia, Bulgaria; arianalangari@abv.bg (A.L.); miroslava.ilieva.ivanova@gmail.com (M.I.)
[3] Institute of Biophysics and Biomedical Engineering, Bulgarian Academy of Sciences, "Acad. G. Bontchev" Str. 21, 1113 Sofia, Bulgaria; sashka.b.krumova@gmail.com
[4] National Specialized Hospital for Active Treatment of Hematological Diseases, Zdrave Str. 2, 1756 Sofia, Bulgaria; l.garcheva@hematology.bg (L.G.); m.genova@hematology.bg (M.G.)
* Correspondence: todinova@abv.bg
† These authors contributed equally to this work.

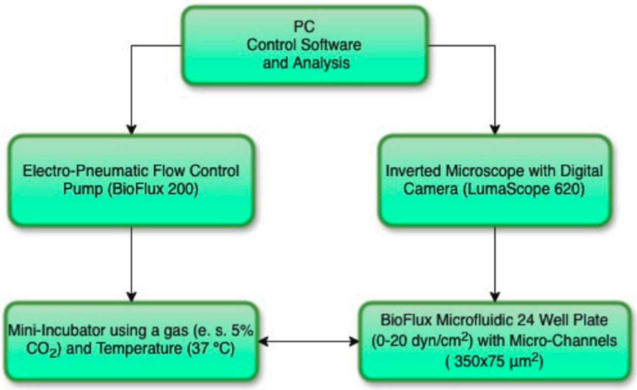

Figure S1. Schematic of the microfluidic system used in this study. The system integrates a BioFlux 200 flow control pump, LumaScope 620 inverted fluorescence microscope with camera, BioFlux 24-well plates (350 × 75 μm² channels, 0–20 dyn/cm²), a mini-incubator (37 °C, 5% CO_2), and PC-based control/analysis software.

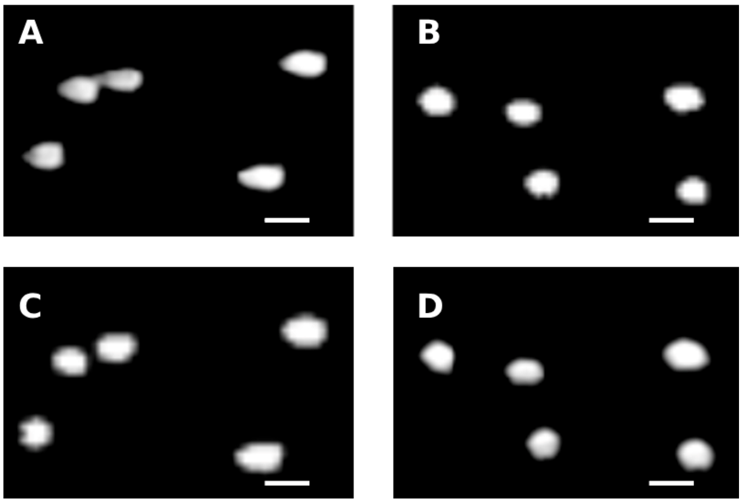

Figure S2. Representative images of deformed RBCs from healthy controls (A, C) and patients with CLL (B, D), analyzed using LabVIEW-based software (A, B) and Python-based software (C, D) under representative flow conditions (268 s^{-1}). The images illustrate the characteristic deformation of RBCs within the microfluidic channel. Scale bar – 10 μm.

Table S1. Number of analyzed RBCs per shear rate in Healthy controls and CLL samples.

**Sample**	**Shear Rate (s^{-1})**						
	**89**	**178**	**268**	**357**	**446**	**535**	**625**
Healthy controls							
H1	296	145	89	79	60	32	25
H2	242	202	112	110	84	46	36
H3	346	282	141	116	83	58	41
H4	308	230	220	200	154	72	53
H5	281	186	166	153	109	86	42
H6	322	245	163	123	102	68	47
H7	137	94	76	68	56	45	33
H8	178	123	102	78	45	28	28
H9	209	174	143	126	77	44	42
H10	226	175	151	116	81	57	33
H11	195	143	114	69	54	41	21
H12	194	169	140	102	96	69	29
H13	161	132	87	58	33	17	
CLL							
CLL1	157	128	107	91	78	64	36
CLL2	192	155	133	124	135	119	96
CLL3	174	135	86	70	61	49	32
CLL4	195	171	143	123	78	44	36
CLL5	190	205	173	137	62	32	19
CLL6	250	247	210	142	85	31	24
CLL7	250	219	137	85	78	42	31
CLL8	188	178	184	160	108	65	32
CLL9	238	132	106	94	54	30	24
